# Comprehensive DNA barcode coverage of North American birds

**DOI:** 10.1111/j.1471-8286.2007.01670.x

**Published:** 2007-07

**Authors:** KEVIN C R KERR, MARK Y STOECKLE, CARLA J DOVE, LEE A WEIGT, CHARLES M FRANCIS, PAUL D N HEBERT

**Affiliations:** * Department of Integrative Biology, Biodiversity Institute of Ontario, University of Guelph Guelph, Ontario, Canada N1G 2W1; † Program for the Human Environment, The Rockefeller University New York, NY 10021, USA; ‡ Smithsonian Institution, National Museum of Natural History Washington, DC 20013-7012, USA; § National Wildlife Research Centre, Canadian Wildlife Service, Environment Canada Ottawa, Ontario, Canada K1A 0H3

**Keywords:** Aves, cryptic species, cytochrome *c* oxidase, DNA barcoding, intraspecific mitochondrial variation, selective sweeps

## Abstract

DNA barcoding seeks to assemble a standardized reference library for DNA-based identification of eukaryotic species. The utility and limitations of this approach need to be tested on well-characterized taxonomic assemblages. Here we provide a comprehensive DNA barcode analysis for North American birds including 643 species representing 93% of the breeding and pelagic avifauna of the USA and Canada. Most (94%) species possess distinct barcode clusters, with average neighbour-joining bootstrap support of 98%. In the remaining 6%, barcode clusters correspond to small sets of closely related species, most of which hybridize regularly. Fifteen (2%) currently recognized species are comprised of two distinct barcode clusters, many of which may represent cryptic species. Intraspecific variation is weakly related to census population size and species age. This study confirms that DNA barcoding can be effectively applied across the geographical and taxonomic expanse of North American birds. The consistent finding of constrained intraspecific mitochondrial variation in this large assemblage of species supports the emerging view that selective sweeps limit mitochondrial diversity.

Mitochondrial DNA (mtDNA) analysis has been employed in the evolutionary study of animal species for more than 30 years (Brown *et al*. 1979; Mindell *et al*. 1997; Avise & Walker 1999). Its higher mutation rate and lower effective population size than nuclear DNA make mtDNA a powerful tool to probe for evidence of reproductive isolation between lineages. This fact provoked a proposal to standardize DNA-based species identification by analysing a uniform segment of the mitochondrial genome. With this approach, a library of sequences from taxonomically verified voucher specimens serve as DNA identifiers for species, in short, DNA barcodes (Hebert *et al*. 2003). For animals, research has focused on a 648-bp segment of the mitochondrial gene cytochrome *c* oxidase I (COI), which can be readily recovered from diverse species with a limited set of primers. DNA barcoding translates expert taxonomic knowledge of diagnostic morphologic characters into a widely accessible format, DNA sequences, enabling more people to identify specimens. In addition to assigning specimens to known species, DNA barcoding can speed the discovery of new species, as large sequence differences in animal mtDNA generally signal species status.

For this approach to be effective, it must be possible to distinguish between intraspecific and interspecific mtDNA variation. Pseudogenes, retention of ancestral polymorphisms, hybridization, and the idiosyncrasies of mtDNA inheritance pose potential difficulties (Benasson *et al*. 2001; Moritz & Cicero 2004; Thalman *et al*. 2004; Will *et al*. 2005). The simplest test is whether genetic distances within species are less than those between species. Surprisingly, 23% of 2319 animal species failed this test in one review (Funk & Omland 2003), implying that mitochondrial gene sequences do not reliably capture species boundaries. However, the published studies that formed the basis for this estimate may be biased towards exceptional situations and groups in need of taxonomic revision, as further investigations on several vertebrate and invertebrate groups have shown that COI barcodes distinguish more than 95% of species (Ward *et al*. 2005; Hajibabaei *et al*. 2006).

Because birds have been the subjects of particularly intensive taxonomic analysis, they provide an excellent opportunity to test the efficacy of barcode-based species delimitation. With most recent species splits stemming from genetic studies, avian taxonomy could, in turn, benefit from a broad-scale genetic survey. In a preliminary survey of 260 North American bird species, COI sequence variation between species was generally much greater than that within species, and no two species shared barcodes (Hebert *et al*. 2004). As a result, COI sequence information enabled assignment of specimens to known species. Four of 120 species (3%) studied in greater detail contained two distinct barcode clusters, which appeared to reflect cryptic species, a conclusion supported by observations of subtle differences in song and morphology for three of the four cases (Rohwer 1976; Kroodsma 1989; Sibley & Monroe 1990). To test these results more stringently, we increase taxon coverage and sample sizes in this study, applying DNA barcoding to examine the taxonomic status of 643 species, representing 93% of the breeding and pelagic bird species from the USA and Canada (Fig. 1).

**Fig. 1:**
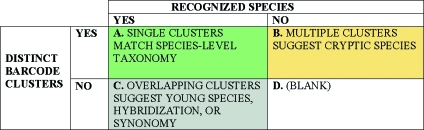
Comparing barcode sequence clusters with species-level taxonomy. Categories A–C are described in the figure; by definition, all potential splits recognized by barcoding have distinct barcodes, so D is blank.

## Materials and methods

Most analytic methods followed those described in the earlier study (Hebert *et al*. 2004). DNA sources for this study included frozen tissue samples (muscle, liver, or blood), most of which were obtained from specimens with vouchers housed in museum collections. In addition to tissue samples, feathers (breast feathers or retrices) freshly collected at bird banding stations at six locales (Ontario, New Brunswick, Nova Scotia, Yukon, North Carolina, Tennessee) were analysed. Feather samples were stored in a dark, dry location at room temperature.

DNA extraction, polymerase chain reaction (PCR), and sequencing reactions were performed at either the University of Guelph or the Smithsonian Institution. DNA was isolated using DryRelease (see Hajibabaei *et al*. 2005), QIAGEN DNeasy tissue extraction kit (QIAGEN), or the NucleoSpin96 tissue kit (Machery-Nagel). Feather samples were processed using the former method exclusively. PCR predominantly utilized a single primer pair: BirdF1 (TTCTCCAACCACAAAGACATT GGCAC) and BirdR1 (ACGTGGGAGATAATTCCAAATCCTG). If amplification was unsuccessful, an alternate forward primer, FalcoFA (TCAACAAACCACAAAGACATCGGCAC), or reverse primers, BirdR2 (ACTACATGTGAGATGATTCCG AATCCAG) and VertebrateR1 (TAGACTTCTGGGTGGCCAAAGAATCA), were employed. All reactions were run under the following thermal cycle program: 1 min at 94 °C followed by six cycles of 1 min at 94 °C, 1.5 min at 45 °C, and 1.5 min at 72 °C, followed in turn by 35 cycles of 1 min at 94 °C, 1.5 min at 55 °C, and 1.5 min at 72 °C, and finally 5 min at 72 °C. Forty-five cycles were run in place of 35 for DNA extracted from feather samples to compensate for lower yields of DNA. PCR products were visualized on precast 2% agarose gels using the E-gel 96 system (Invitrogen). PCR products were bidirectionally sequenced on an ABI 3100, 3130, or 3730. Contigs were assembled from forward and reverse reads using sequencher, version 4.5 (Gene Codes).

Specimen and collection data, sequences, and trace files are provided in the container project ‘Birds of North America Phase 2’ at http://www.barcodinglife.org. A Kimura 2-parameter distance metric was employed for sequence comparisons (Kimura 1980), genetic distances were calculated using the bold Management & Analysis System (http://www.barcodinglife.org), bootstrap analysis was performed with 1000 replicates using mega, version 3.1 (Kumar *et al*. 2004), and scatter and box plots were generated with sigmaplot 8.02 (spss). All new sequences have been deposited in GenBank under Accession nos DQ432694 to DQ433261, DQ433274 to DQ433846, and DQ434243 to DQ434805, while sequences from the earlier study (Hebert *et al*. 2004) are deposited in GenBank under Accession nos AY666171 to AY666596.

## Results

A standard set of primers amplified the target region of COI from all but one of 643 species. These taxa included representatives from 19 (70%) of the 27 extant orders of birds, distributed among 71 families and 286 genera (see Table S1, Supplementary material). Together with the 438 specimens analysed in the earlier study, we obtained COI sequences from 2590 individuals, 70% from vouchered specimens held in museum collections. The mean length of the products sequenced was 658 bp. We analysed multiple individuals (average = 4.1, range = 2–125) from 546 (85%) of the 642 species, including five or more individuals from 211 species (33%). In most cases, conspecific specimens derived from widely separated sites (Birds of North America Phase 2 project at http://www.barcodinglife.org).

We detected presumptive pseudogenes in approximately 5% of the specimens. Because these were generally short, approximately 100–200 nucleotides, complete barcode sequences could be recovered with bidirectional sequencing. One presumptive pseudogene corresponding to the full-length barcode sequence was detected in three tyrannid flycatcher specimens (0.1%). Overall, pseudogenes were not an important limit to recovery of COI sequences.

**Fig. 2:**
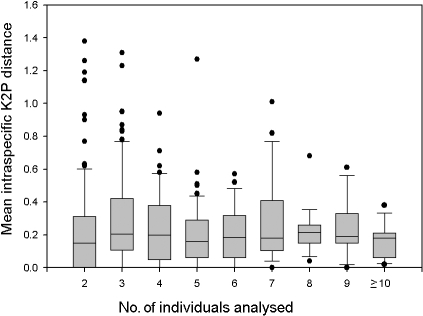
Mean intraspecific variation according to number of individuals analysed. Boxes indicate mean, 25th and 75th percentile; bars, 10th and 90th percentile; and dots, values above or below 90th or 10th percentile, respectively.

**Fig. 3:**
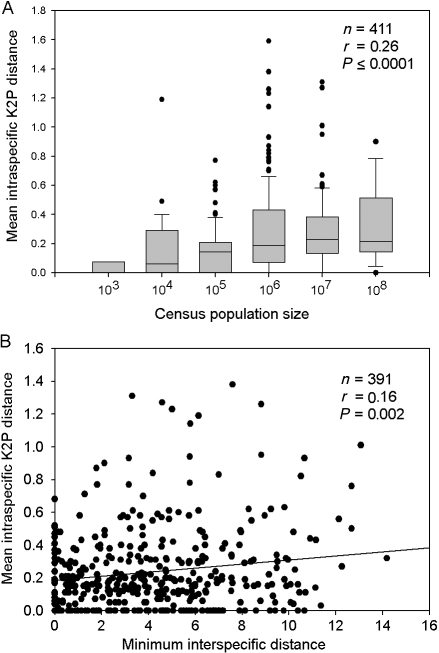
Intraspecific distance, population size, and apparent species age. (A) Linear regression of mean intraspecific distance and log_10_ census population size. For illustration purposes, a box plot was generated as described in legend to Fig. 2. (B) Linear regression of mean intraspecific COI distance compared with apparent species age, as indicated by minimum interspecific Kimura 2-parameter (K2P) distance to nearest congeneric relative.

**Fig. 4:**
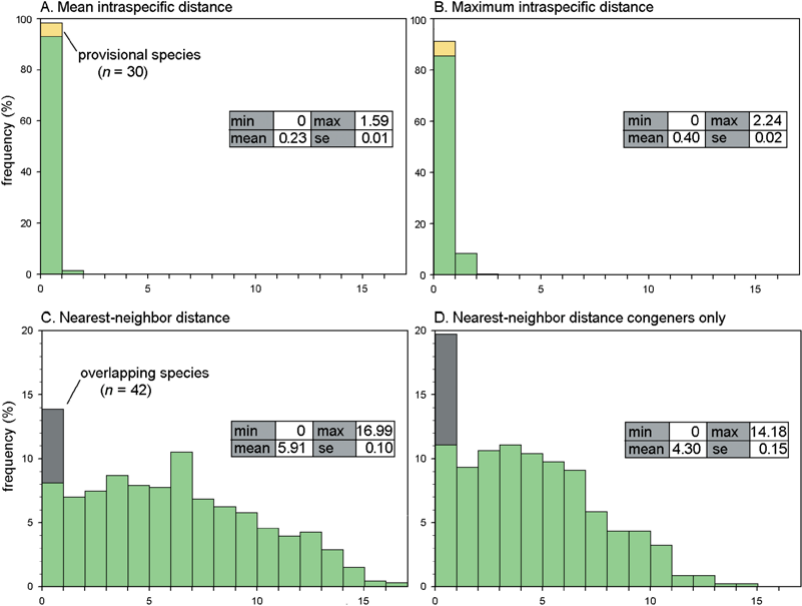
Intraspecific and nearest-neighbour distances in North American birds. Applying these measures to the data set in the preliminary study (2) gave mean values of 0.24, 0.27, 8.02, and 5.86 for A–D, respectively.

Average intraspecific variation was unrelated to the number of individuals analysed, suggesting there was representative sampling (Fig. 2). Within the low and narrow band of intraspecific variation, there was a weak relationship to census population size, which ranges from a few thousand to over 300 million individuals (Fig. 3) (Wetlands International 2002; Rich *et al*. 2004). Intraspecific mitochondrial variation was only weakly associated with apparent species age (Fig. 3). The earlier North American bird study measured *mean congeneric distance*, the average distance among all congeneric relatives. To more stringently test the discriminatory power of COI barcodes, the present study examined *nearest-neighbour distance*, the minimum genetic distance between a species and its closest congeneric relative. Nearest-neighbour distance averaged 4.3%, 19-fold higher than the mean within species and 11-fold higher than the average maximum intraspecific distance (Fig. 4). Including all species may give a more representative picture, as generic assignments may be incorrect, and 10% of birds are the sole members of their genus; in this case, average nearest-neighbour distance was higher at 5.9% (Fig. 4).

Levels of sequence difference varied across families: 35% of ducks, geese, and swans (Anatidae) showed nearest- neighbour differences of 1% or less, whereas all sandpipers (Scolopacidae), plovers (Charadriidae), and owls (Strigidae) had nearest-neighbour distances greater than 1%. COI barcodes separated 20 of the 23 taxonomic splits recognized in North American birds over the past 25 years with nearest-neighbour distances ranging from 0.3 to 6.0% (see Table S2, Supplementary material). Average bootstrap support for species nodes with multiple individuals was 97.8%. As expected, bootstrap values were lower among the most closely related species, averaging 79.8% for species with nearest-neighbour distances less than 1%, but 99.5% for species with distances above 1%.

Forty-two species (6.4%) shared sequences or had clusters of sequences overlapping with those of another species, including 14 pairs, two triplets, and one set of eight species (Table 1). The pattern of COI variation within these sets of overlapping species was indistinguishable from variation within single species, with the exception of mallards and black ducks, which are known to harbour two distinct mitochondrial lineages (Avise *et al*. 1990). By contrast, we detected 15 other species with intraspecific distances greater than 2.5% (Table 2); each contained two distinct sequence clusters typically comprised of individuals from different geographical areas. These clusters may represent cryptic species. Treating these provisional species as distinct, average within-species variation for the COI barcode region was 0.23%.

**Table 2:** Provisional species. Provisional splits of recognized species with intraspecific distances above 2.5% threshold (*) identified in earlier study (Hebert *et al*. 2004); (†) prior research supports split (see Table S3, Supplementary material); (‡) prior research cites genetic division but does not support species split (citations provided in this table). Bootstrap support for provisional species clusters are shown

	Common name	Scientific name	Maximum intraspecific distance	Bootstrap	Citation (if applicable)
1	Northern fulmar	*Fulmaris glacialis*	3.1	100/—	
2	Solitary sandpiper*	*Tringa solitaria*	5.4	100/100	
3	Western screech owl	*Megascops kennicottii*	3.1	100/100	
4	Warbling vireo*†	*Vireo gilvus*	4.0	100/99	Sibley & Monroe 1990
5	Mexican jay†	*Aphelocoma ultramarina*	5.3	100/—	Rice *et al*. 2003
6	Western scrub-jay†	*Aphelocoma californica*	3.2	77/—	Rice *et al*. 2003
7	Common raven†	*Corvus corax*	4.3	100/92	Omland *et al*. 2000
8	Mountain chickadee†	*Poecile gambeli*	3.7	100/100	Gill *et al*. 1993
9	Bushtit	*Psaltriparus minimus*	3.6	100/100	
10	Winter wren†	*Troglodytes troglodytes*	6.4	100/100	Drovetski 2004
11	Marsh wren*†	*Cistothorus palustris*	7.9	100/100	Kroodsma 1989
12	Bewick's wren	*Thyromanes bewickii*	4.8	100/100	
13	Hermit thrush	*Catharus guttatus*	3.2	100/100	
14	Curve-billed thrasher†	*Toxostoma curvirostre*	7.4	100/—	Zink & Blackwell-Rago 2000
15	Eastern meadowlark*†	*Sturnella magna*	4.6	100/100	Rohwer 1976

**Table 1:** Species with overlapping barcode clusters. The per cent similarity between related species (calculated using a Kimura 2-parameter distance metric) is provided

	Order	Common name	Scientific name	*n*	% similarity
1	Anseriformes	Snow goose	*Chen caerulescens*	5	99.8
		Ross's goose	*Chen rossii*	2	
2		American Black duck	*Anas rubripes*	8	
		Mallard	*Anas platyrhynchos*	8	99.4
		Mottled duck	*Anas fulvigula*	1	
3		Blue-winged teal	*Anas discors*	8	100.0
		Cinnamon teal	*Anas cyanoptera*	2	
4		King eider	*Somateria spectabilis*	5	99.7
		Common eider	*Scomateria mollissima*	1	
5	Galliformes	Sharp-tailed grouse	*Tympanuchus phasianellus*	3	99.7
		Greater prairie-chicken	*Tympanuchus cupido*	1	
		Lesser prairie-chicken	*Tympanuchus pallidicinctus*	5	
6	Podicipediformes	Western grebe	*Aechmophorus occidentalis*	2	99.7
		Clark's grebe	*Aechmophorus clarkii*	2	
7	Charadriiformes	Laughing gull	*Larus atricilla*	8	99.3
		Franklin's gull	*Larus pipixcan*	4	
8		California gull	*Larus californicus*	5	99.8
		Herring gull	*Larus argentatus*	7	
		Thayer's gull	*Larus thayeri*	4	
		Iceland gull	*Larus glaucoides*	1	
		Lesser black-backed gull	*Larus fuscus*	5	
		Western gull	*Larus occidentalis*	4	
		Glaucous-winged gull	*Larus glaucescens*	4	
		Glaucous gull	*Larus hyperboreus*	4	
9	Piciformes	Red-naped sapsucker	*Sphyrapicus nuchalis*	5	99.4
		Red-breasted sapsucker	*Sphyrapicus ruber*	6	
10	Passeriformes	Black-billed magpie	*Pica hudsonia*	3	99.6
		Yellow-billed magpie	*Pica nuttalli*	3	
11		American crow	*Corvus brachyrhynchos*	3	99.5
		Northwestern crow	*Corvus caurinus*	4	
12		Townsend's warbler	*Dendroica townsendi*	6	99.5
		Hermit warbler	*Dendroica occidentalis*	5	
13		Golden-crowned sparrow	*Zonotrichia leucophrys*	8	99.7
		White-crowned sparrow	*Zonotrichia atricapilla*	3	
14		Dark-eyed junco	*Junco hyemalis*	24	99.7
		Yellow-eyed junco	*Junco phaeonotus*	3	
15		Snow bunting	*Plectrophenax nivalis*	2	99.9
		McKay's bunting	*Plectrophenax hyperboreus*	1	
16		Great-tailed grackle	*Quiscalis mexicanus*	11	99.2
		Boat-tailed grackle	*Quiscalis major*	6	
17		Common redpoll	*Carduelis flammea*	2	99.7
		Hoary redpoll	*Carduelis hornemanni*	5	

## Discussion

The present study has reaffirmed that most North American bird species correspond to a single, tightly cohesive array of barcode sequences that are distinct from those of any other species. However, 15 species include two distinct barcode clusters, while 42 other species possess barcode sequences that are shared or overlap with those of other species. What explains these exceptional cases?

Cases of deep barcode divergence within what are thought to be single species generally indicate cryptic taxa (Moritz 1994; Meyer & Paulay 2005). Our screen for provisional splits in species, employing a threshold that was 10× higher than the mean intraspecific variation, revealed 15 cases. Results from a thresholding approach must be interpreted with caution and are best used to flag species in need of further research. Significantly, most of these hypothesized splits are supported by prior taxonomic work (Table 2). In total, nine of our 15 cases have been previously cited; eight have been proposed to represent species pairs, the exceptional case being the northern raven (Omland *et al*. 2000). Some of the species yield additional lineages when non-North American populations are included; for example, six lineages in total are suggested for the winter wren (Drovetski *et al*. 2004).

Regarding the 17 sets of species with overlapping barcodes, three processes may account for these findings. First, some may be recently diverged sister taxa where COI has not yet accumulated sequence differences. In such cases, more extensive sequence information might allow resolution. Second, these taxa may share mtDNA because of hybridization. Most of our species sets with overlapping barcodes hybridize at least occasionally; many show extensive hybridization and produce fertile F1 hybrid offspring. Examples include snow goose and Ross's goose (Cook *et al*. 1995); blue-winged and cinnamon teal (Bolen 1979); mallard, mottled, and black ducks (McCracken *et al*. 2001); sharp-tailed grouse and greater prairie-chicken (Sparling 1980); red-naped and red-breasted sapsuckers (Johnson & Johnson 1985); Townsend's and hermit warblers (Morrison & Hardy 1983); and the eight species of large white-headed gulls (California, Glaucous, Glaucous-winged, Herring, Iceland, lesser black-backed, western, Thayer's; Olsen & Larsson 2004). These taxa may be in the indeterminate zone between differentiated populations and distinct species (de Queiroz 2005), or well-formed species that are losing genetic identity due to secondary contact and hybridization. Third, some of the pairs with overlapping barcodes may be a single species (Johnston 1961).

Although there is an abundance of *subspecific* assignments in North American birds — 5.5 per species according to one survey — many do not show any evidence of genetic divergence (Zink 2004). Barcode analyses can serve as a quick screening tool for those lineages with deep genetic divergence, aiding detection of overlooked species. In fact, all past barcode surveys have identified new taxonomic units, either as named species, provisional species, evolutionarily significant units (ESUs), or molecular operational taxonomic units (MOTUs) in 4–40% of the species examined (Meyer & Paulay 2005; Monaghan *et al*. 2005; Saunders 2005; Smith *et al*. 2005; Hajibabaei *et al*. 2006; Scheffer *et al*. 2006). These results suggest that ‘an iterative process of DNA barcoding … followed by taxonomic study’ will be a productive path to cataloguing biodiversity (Barber & Boyce 2006). In the present study, most provisional species were small to medium-sized, plainly coloured birds, whereas most species with overlapping barcodes were large and/or brightly coloured, which might reflect a natural taxonomic tendency toward undersplitting inconspicuous birds and/or oversplitting more conspicuous species.

Over 30 years ago, Richard Lewontin concluded that intraspecific variation is tightly constrained and recognized that both genetic drift and natural selection offer possible explanations for this fact (Lewontin 1974). Under genetic drift, recent population bottlenecks could account for low intraspecific variation. It might be argued that the low levels of mitochondrial variation detected in our study reflect the unique history of North American birds, most of which have expanded into their present ranges from smaller populations following retreat of glaciers. However, restricted intraspecific mitochondrial variation also exists in many vertebrate and invertebrate species from tropical, temperate, marine, and terrestrial environments (Barrowclough & Shields 1984; Bucklin & Wiebe 1998; Meyer & Paulay 2005; Saunders 2005; Hajibabaei *et al*. 2006), implying a more general explanation. Effective population size for nuclear genes can reach an asymptotic limit due to linkage; this effect is strongest for organisms with large genomes, with the result that the effective population size of vertebrates might not exceed 10^4^ (Gillespie 2000; Lynch *et al*. 2006). Although not directly applicable to mitochondria, this effect does reveal the complexities of estimating effective population sizes and predicting the role of drift in scouring variation.

Low mitochondrial variation might alternatively (or additionally) reflect recurrent selective sweeps; repeated diffusions of new, selectively favoured variants across the breeding range of a species could purge mitochondrial diversity. Although 98% of the nucleotide differences in COI barcode sequences in our study between nearest neighbours were synonymous, selection on any nucleotide position in the mitochondrial genome would result in the loss of variation in the barcode region because mtDNA is inherited as a single linkage group, due to its asexual transmission. Mutations in nuclear or mitochondrial loci important in nuclear-mitochondrial co-adaptation might be particularly important (Catalano *et al*. 2006). A recent analysis of patterns of substitution in nuclear and mtDNA concluded that reduced mitochondrial diversity in animals is due to selective sweeps (Bazin *et al*. 2006). Although these authors found no correlation between census population size and intraspecific mitochondrial variation, the range of variation was less than expected given census population sizes. This latter finding, together with our results showing trends toward increased diversity in larger populations and older species, imply that genetic drift does influence mitochondrial variation, but only weakly.

Most researchers agree that species are a key unit of biological systems, but quarrel about how best to define them. Hence, theoretical and operational species concepts proliferate, each emphasizing different aspects of present-day biology and evolutionary history (Wheeler & Meirer 2000). Some believe that a basic taxonomic unit does not exist, instead viewing species as a convenient taxonomic construct, ‘an arbitrary cut-off somewhere along a branch in the tree of life’ (Mishler & Shapley 2004). The tight clustering of mtDNA sequences within species observed in our study not only bolsters the view that species are fundamental biological units, but also reveals that their identification is usually uncomplicated.

In summary, most North American bird species appear to have a similar genetic structure, each being a single tight cluster of mtDNA variants distinct from the clusters of closely related species. High bootstrap support for species nodes in this study and in other animal groups suggests neighbour-joining analysis of COI barcode sequences will be widely effective (Ward *et al*. 2005; Hajibabaei *et al*. 2006). The few species with higher intraspecific diversity were comprised of two such clusters, many of which appear to represent cryptic species. It seems likely that further study will reveal additional lineages within some species, but leave unchanged the underlying pattern of segregation of mitochondrial diversity into distinct clusters (Zink 2004). Together these observations imply a general constraint on mitochondrial diversity in birds.

## Supplementary material

The supplementary material is available from http://www.blackwellpublishing.com/products/journals/suppmat/MEN/MEN1670/MEN1670sm.htm

Table S1 Species and number of individuals analysed

Table S2 COI barcode distances between recent taxonomic splits in North American birds
