# Supplementary material for: Comprehensive DNA barcode coverage of North American birds
Source: Mol Ecol Notes. 2007 Jul;7(4):535–43. doi: 10.1111/j.1471-8286.2007.01670.x (PMC2259444; doi:10.1111/j.1471-8286.2007.01670.x)
Supplement: Table S1 — Species and number of individuals analysed [file men0007-0535-SD1.doc]

**Electronic Supplementary Material**

**Table S1.** Species and number of individuals analyzed.

**Table S2.** COI barcode distances between recent taxonomic splits in North American birds.

**Supporting Information**

**Table S1.** Species and number of individuals analyzed, arranged by taxonomic order according to American Ornithologists’ Union Check-list. COI barcode sequences were obtained from 642 of 643 species analyzed (exception was Sooty Shearwater, *Puffinis griseus*). Provisional species are designated PS-1, PS-2 following species name.

|  | Common Name | Scientific Name | n |
| --- | --- | --- | --- |
| 1 | Black-bellied Whistling-Duck | *Dendrocygna autumnalis* | 1 |
| 2 | Greater White-fronted Goose | *Anser albifrons* | 3 |
| 3 | Emperor Goose | *Chen canagica* | 1 |
| 4 | Snow Goose | *Chen caerulescens* | 5 |
| 5 | Ross’s Goose | *Chen rossii* | 2 |
| 6 | Brant | *Branta bernicla* | 10 |
| 7 | Barnacle Goose | *Branta leucopsis* | 1 |
| 8 | Cackling Goose | *Branta hutchinsi* | 29 |
| 9 | Canada Goose | *Branta canadensis* | 125 |
| 10 | Mute Swan | *Cygnus olor* | 2 |
| 11 | Trumpeter Swan | *Cygnus buccinator* | 2 |
| 12 | Tundra Swan | *Cygnus columbianus* | 2 |
| 13 | Muscovy Duck | *Cairina moschata* | 2 |
| 14 | Wood Duck | *Aix sponsa* | 9 |
| 15 | Gadwall | *Anas strepera* | 6 |
| 16 | American Wigeon | *Anas americana* | 8 |
| 17 | American Black Duck | *Anas rubripes* | 8 |
| 18 | Mallard | *Anas platyrhynchos* | 8 |
| 19 | Mottled Duck | *Anas fulvigula* | 1 |
| 20 | Blue-winged Teal | *Anas discors* | 8 |
| 21 | Cinnamon Teal | *Anas cyanoptera* | 2 |
| 22 | Northern Shoveler | *Anas clypeata* | 8 |
| 23 | Northern Pintail | *Anas acuta* | 7 |
| 24 | Green-winged Teal | *Anas crecca* | 9 |
| 25 | Canvasback | *Aythya valisineria* | 6 |
| 26 | Redhead | *Aythya americana* | 10 |
| 27 | Tufted Duck | *Aythya fuligula* | 3 |
| 28 | Ring-necked Duck | *Aythya collaris* | 10 |
| 29 | Greater Scaup | *Aythya marila* | 10 |
| 30 | Lesser Scaup | *Aythya affinis* | 10 |
| 31 | Steller’s Eider | *Polysticta stelleri* | 2 |
| 32 | Spectacled Eider | *Somateria fischeri* | 7 |
| 33 | King Eider | *Somateria spectabilis* | 5 |
| 34 | Common Eider | *Somateria mollissima* | 10 |
| 35 | Harlequin Duck | *Histrionicus histrionicus* | 3 |
| 36 | Surf Scoter | *Melanitta perspicillata* | 2 |
| 37 | White-winged Scoter | *Melanitta fusca* | 5 |
| 38 | Black Scoter | *Melanitta nigra* | 7 |
| 39 | Long-tailed Duck | *Clangula hyemalis* | 9 |
| 40 | Bufflehead | *Bucephala albeola* | 8 |
| 41 | Common Goldeneye | *Bucephala clangula* | 7 |
| 42 | Barrow’s Goldeneye | *Bucephala islandica* | 11 |
| 43 | Hooded Merganser | *Lophodytes cucullatus* | 9 |
| 44 | Common Merganser | *Mergus merganser* | 9 |
| 45 | Red-breasted Merganser | *Mergus serrator* | 6 |
| 46 | Ruddy Duck | *Oxyura jamaicensis* | 6 |
| 47 | Chukar | *Alectoris chukar* | 2 |
| 48 | Gray Partridge | *Perdix perdix* | 6 |
| 49 | Ring-necked Pheasant | *Phasianus colchicus* | 2 |
| 50 | Ruffed Grouse | *Bonasa umbellus* | 8 |
| 51 | Greater Sage-Grouse | *Centrocercus urophasianus* | 5 |
| 52 | Gunnison Sage-Grouse | *Centrocercus minimus* | 2 |
| 53 | Spruce Grouse | *Falcipennis canadensis* | 4 |
| 54 | Willow Ptarmigan | *Lagopus lagopus* | 5 |
| 55 | Rock Ptarmigan | *Lagopus mutus* | 21 |
| 56 | White-tailed Ptarmigan | *Lagopus leucurus* | 5 |
| 57 | Dusky Grouse | *Dendragapus obscurus* | 4 |
| 58 | Sooty Grouse | *Dendragapus fuliginosus* | 2 |
| 59 | Sharp-tailed Grouse | *Tympanuchus phasianellus* | 3 |
| 60 | Greater Prairie-Chicken | *Tympanuchus cupido* | 1 |
| 61 | Lesser Prairie-Chicken | *Tympanuchus pallidicinctus* | 5 |
| 62 | Wild Turkey | *Meleagris gallopavo* | 3 |
| 63 | Mountain Quail | *Oreortyx pictus* | 4 |
| 64 | Scaled Quail | *Callipepla squamata* | 2 |
| 65 | California Quail | *Callipepla californica* | 1 |
| 66 | Gambel’s Quail | *Callipepla gambelii* | 2 |
| 67 | Northern Bobwhite | *Colinus virginianus* | 3 |
| 68 | Red-throated Loon | *Gavia stellata* | 2 |
| 69 | Arctic Loon | *Gavia arctica* | 1 |
| 70 | Pacific Loon | *Gavia pacifica* | 6 |
| 71 | Common Loon | *Gavia immer* | 3 |
| 72 | Yellow-billed Loon | *Gavia adamsii* | 6 |
| 73 | Least Grebe | *Tachybaptus dominicus* | 1 |
| 74 | Pied-billed Grebe | *Podilymbus podiceps* | 5 |
| 75 | Horned Grebe | *Podiceps auritus* | 2 |
| 76 | Red-necked Grebe | *Podiceps grisegena* | 3 |
| 77 | Eared Grebe | *Podiceps nigricollis* | 3 |
| 78 | Western Grebe | *Aechmophorus occidentalis* | 2 |
| 79 | Clark’s Grebe | *Aechmophorus clarkii* | 2 |
| 80 | Laysan Albatross | *Phoebastria immutabilis* | 4 |
| 81 | Black-footed Albatross | *Phoebastria nigripes* | 3 |
| 82 | Northern Fulmar PS-1 | *Fulmarus glacialis PS-1* | 1 |
| 83 | Northern Fulmar PS-2 | *Fulmarus glacialis PS-2* | 3 |
| 84 | Black-capped Petrel | *Pterodroma hasitata* | 2 |
| 85 | Cory’s Shearwater | *Calonectris diomedea* | 2 |
| 86 | Pink-footed Shearwater | *Puffinus creatopus* | 3 |
| 87 | Flesh-footed Shearwater | *Puffinus carneipes* | 5 |
| 88 | Greater Shearwater | *Puffinus gravis* | 1 |
| 89 | Wedge-tailed Shearwater | *Puffinus pacificus* | 7 |
| 90 | Buller's Shearwater | *Puffinus bulleri* | 6 |
| 91 | Short-tailed Shearwater | *Puffinus tenuirostris* | 6 |
| 92 | Manx Shearwater | *Puffinus puffinus* | 1 |
| 93 | Audubon’s Shearwater | *Puffinus lherminieri* | 1 |
| 94 | Wilson’s Storm-Petrel | *Oceanites oceanicus* | 2 |
| 95 | Fork-tailed Storm-Petrel | *Oceanodroma furcata* | 2 |
| 96 | Leach’s Storm-Petrel | *Oceanodroma leucorhoa* | 4 |
| 97 | Ashy Storm-Petrel | *Oceanodroma homochroa* | 1 |
| 98 | Band-rumped Storm-Petrel | *Oceanodroma castro* | 1 |
| 99 | Masked Booby | *Sula dactylatra* | 1 |
| 100 | Northern Gannet | *Morus bassanus* | 4 |
| 101 | American White Pelican | *Pelecanus erythrorhynchos* | 4 |
| 102 | Brown Pelican | *Pelecanus occidentalis* | 2 |
| 103 | Brandt’s Cormorant | *Phalacrocorax penicillatus* | 5 |
| 104 | Neotropic Cormorant | *Phalacrocorax brasilianus* | 1 |
| 105 | Double-crested Cormorant | *Phalacrocorax auritus* | 4 |
| 106 | Great Cormorant | *Phalacrocorax carbo* | 4 |
| 107 | Red-faced Cormorant | *Phalacrocorax urile* | 1 |
| 108 | Pelagic Cormorant | *Phalacrocorax pelagicus* | 9 |
| 109 | Anhinga | *Anhinga anhinga* | 3 |
| 110 | Magnificent Frigatebird | *Fregata magnificens* | 2 |
| 111 | American Bittern | *Botaurus lentiginosus* | 3 |
| 112 | Least Bittern | *Ixobrychus exilis* | 2 |
| 113 | Great Blue Heron | *Ardea herodias* | 5 |
| 114 | Great Egret | *Ardea alba* | 3 |
| 115 | Snowy Egret | *Egretta thula* | 1 |
| 116 | Little Blue Heron | *Egretta caerulea* | 2 |
| 117 | Tricolored Heron | *Egretta tricolor* | 3 |
| 118 | Reddish Egret | *Egretta rufescens* | 1 |
| 119 | Cattle Egret | *Bubulcus ibis* | 4 |
| 120 | Green Heron | *Butorides virescens* | 2 |
| 121 | Black-crowned Night-Heron | *Nycticorax nycticorax* | 1 |
| 122 | Yellow-crowned Night-Heron | *Nyctanassa violacea* | 1 |
| 123 | White Ibis | *Eudocimus albus* | 6 |
| 124 | Glossy Ibis | *Plegadis falcinellus* | 1 |
| 125 | White-faced Ibis | *Plegadis chihi* | 3 |
| 126 | Roseate Spoonbill | *Platalea ajaja* | 2 |
| 127 | Wood Stork | *Mycteria americana* | 2 |
| 128 | Black Vulture | *Coragyps atratus* | 5 |
| 129 | Turkey Vulture | *Cathartes aura* | 4 |
| 130 | Osprey | *Pandion haliaetus* | 5 |
| 131 | Swallow-tailed Kite | *Elanoides forficatus* | 1 |
| 132 | White-tailed Kite | *Elanus leucurus* | 2 |
| 133 | Snail Kite | *Rostrhamus sociabilis* | 2 |
| 134 | Mississippi Kite | *Ictinia mississippiensis* | 1 |
| 135 | Bald Eagle | *Haliaeetus leucocephalus* | 5 |
| 136 | Northern Harrier | *Circus cyaneus* | 4 |
| 137 | Sharp-shinned Hawk | *Accipiter striatus* | 5 |
| 138 | Cooper’s Hawk | *Accipiter cooperii* | 5 |
| 139 | Northern Goshawk | *Accipiter gentilis* | 7 |
| 140 | Gray Hawk | *Asturina nitida* | 2 |
| 141 | Common Black-Hawk | *Buteogallus anthracinus* | 2 |
| 142 | Harris’s Hawk | *Parabuteo unicinctus* | 1 |
| 143 | Red-shouldered Hawk | *Buteo lineatus* | 2 |
| 144 | Broad-winged Hawk | *Buteo platypterus* | 3 |
| 145 | Short-tailed Hawk | *Buteo brachyurus* | 1 |
| 146 | Swainson’s Hawk | *Buteo swainsoni* | 3 |
| 147 | White-tailed Hawk | *Buteo albicaudatus* | 2 |
| 148 | Zone-tailed Hawk | *Buteo albonotatus* | 2 |
| 149 | Red-tailed Hawk | *Buteo jamaicensis* | 1 |
| 150 | Ferruginous Hawk | *Buteo regalis* | 1 |
| 151 | Rough-legged Hawk | *Buteo lagopus* | 2 |
| 152 | Golden Eagle | *Aquila chrysaetos* | 2 |
| 153 | Crested Caracara | *Caracara cheriway* | 1 |
| 154 | American Kestrel | *Falco sparverius* | 5 |
| 155 | Merlin | *Falco columbarius* | 4 |
| 156 | Aplomado Falcon | *Falco femoralis* | 1 |
| 157 | Gyrfalcon | *Falco rusticolus* | 2 |
| 158 | Peregrine Falcon | *Falco peregrinus* | 5 |
| 159 | Prairie Falcon | *Falco mexicanus* | 2 |
| 160 | Yellow Rail | *Coturnicops noveboracensis* | 1 |
| 161 | Black Rail | *Laterallus jamaicensis* | 1 |
| 162 | Clapper Rail | *Rallus longirostris* | 2 |
| 163 | King Rail | *Rallus elegans* | 1 |
| 164 | Virginia Rail | *Rallus limicola* | 3 |
| 165 | Sora | *Porzana carolina* | 3 |
| 166 | Purple Gallinule | *Porphyrio martinica* | 2 |
| 167 | Moorhen | *Gallinula chloropus* | 6 |
| 168 | American Coot | *Fulica americana* | 5 |
| 169 | Limpkin | *Aramus guarauna* | 3 |
| 170 | Sandhill Crane | *Grus canadensis* | 1 |
| 171 | Whooping Crane | *Grus americana* | 4 |
| 172 | Black-bellied Plover | *Pluvialis squatarola* | 3 |
| 173 | American Golden-Plover | *Pluvialis dominica* | 4 |
| 174 | Pacific Golden-Plover | *Pluvialis fulva* | 2 |
| 175 | Mongolian Plover | *Charadrius mongolus* | 1 |
| 176 | Snowy Plover | *Charadrius alexandrinus* | 3 |
| 177 | Wilson’s Plover | *Charadrius wilsonia* | 2 |
| 178 | Common Ringed Plover | *Charadrius hiaticula* | 2 |
| 179 | Semipalmated Plover | *Charadrius semipalmatus* | 5 |
| 180 | Piping Plover | *Charadrius melodus* | 6 |
| 181 | Killdeer | *Charadrius vociferus* | 3 |
| 182 | Mountain Plover | *Charadrius montanus* | 2 |
| 183 | Eurasian Dotterel | *Charadrius morinellus* | 1 |
| 184 | American Oystercatcher | *Haematopus palliatus* | 2 |
| 185 | Black Oystercatcher | *Haematopus bachmani* | 4 |
| 186 | Black-necked Stilt | *Himantopus mexicanus* | 3 |
| 187 | American Avocet | *Recurvirostra americana* | 2 |
| 188 | Northern Jacana | *Jacana spinosa* | 2 |
| 189 | Greater Yellowlegs | *Tringa melanoleuca* | 3 |
| 190 | Lesser Yellowlegs | *Tringa flavipes* | 2 |
| 191 | Wood Sandpiper | *Tringa glareola* | 4 |
| 192 | Solitary Sandpiper PS-1 | *Tringa solitaria PS-1* | 5 |
| 193 | Solitary Sandpiper PS-2 | *Tringa solitaria PS-2* | 5 |
| 194 | Willet | *Catoptrophorus semipalmatus* | 1 |
| 195 | Wandering Tattler | *Heteroscelus incanus* | 1 |
| 196 | Spotted Sandpiper | *Actitis macularia* | 6 |
| 197 | Upland Sandpiper | *Bartramia longicauda* | 3 |
| 198 | Whimbrel | *Numenius phaeopus* | 2 |
| 199 | Bristle-thighed Curlew | *Numenius tahitiensis* | 1 |
| 200 | Long-billed Curlew | *Numenius americanus* | 2 |
| 201 | Hudsonian Godwit | *Limosa haemastica* | 2 |
| 202 | Bar-tailed Godwit | *Limosa lapponica* | 2 |
| 203 | Marbled Godwit | *Limosa fedoa* | 3 |
| 204 | Ruddy Turnstone | *Arenaria interpres* | 4 |
| 205 | Black Turnstone | *Arenaria melanocephala* | 1 |
| 206 | Surfbird | *Aphriza virgata* | 2 |
| 207 | Red Knot | *Calidris canutus* | 2 |
| 208 | Sanderling | *Calidris alba* | 3 |
| 209 | Semipalmated Sandpiper | *Calidris pusilla* | 3 |
| 210 | Western Sandpiper | *Calidris mauri* | 4 |
| 211 | Least Sandpiper | *Calidris minutilla* | 3 |
| 212 | White-rumped Sandpiper | *Calidris fuscicollis* | 3 |
| 213 | Baird’s Sandpiper | *Calidris bairdii* | 2 |
| 214 | Pectoral Sandpiper | *Calidris melanotos* | 3 |
| 215 | Purple Sandpiper | *Calidris maritima* | 1 |
| 216 | Rock Sandpiper | *Calidris ptilocnemis* | 2 |
| 217 | Dunlin | *Calidris alpina* | 3 |
| 218 | Stilt Sandpiper | *Calidris himantopus* | 3 |
| 219 | Buff-breasted Sandpiper | *Tryngites subruficollis* | 1 |
| 220 | Ruff | *Philomachus pugnax* | 2 |
| 221 | Short-billed Dowitcher | *Limnodromus griseus* | 6 |
| 222 | Long-billed Dowitcher | *Limnodromus scolopaceus* | 2 |
| 223 | Wilson’s Snipe | *Gallinago delicata* | 6 |
| 224 | American Woodcock | *Scolopax minor* | 5 |
| 225 | Wilson’s Phalarope | *Phalaropus tricolor* | 2 |
| 226 | Red-necked Phalarope | *Phalaropus lobatus* | 7 |
| 227 | Red Phalarope | *Phalaropus fulicarius* | 2 |
| 228 | Great Skua | *Stercorarius skua* | 2 |
| 229 | Pomarine Jaeger | *Stercorarius pomarinus* | 5 |
| 230 | Parasitic Jaeger | *Stercorarius parasiticus* | 3 |
| 231 | Long-tailed Jaeger | *Stercorarius longicaudus* | 5 |
| 232 | Laughing Gull | *Larus atricilla* | 8 |
| 233 | Franklin’s Gull | *Larus pipixcan* | 5 |
| 234 | Black-headed Gull | *Larus ridibundus* | 5 |
| 235 | Bonaparte’s Gull | *Larus philadelphia* | 4 |
| 236 | Heermann’s Gull | *Larus heermanni* | 3 |
| 237 | Mew Gull | *Larus canus* | 4 |
| 238 | Ring-billed Gull | *Larus delawarensis* | 3 |
| 239 | California Gull | *Larus californicus* | 5 |
| 240 | Herring Gull | *Larus argentatus* | 7 |
| 241 | Thayer’s Gull | *Larus thayeri* | 2 |
| 242 | Iceland Gull | *Larus glaucoides* | 1 |
| 243 | Lesser Black-backed Gull | *Larus fuscus* | 5 |
| 244 | Western Gull | *Larus occidentalis* | 4 |
| 245 | Glaucous-winged Gull | *Larus glaucescens* | 4 |
| 246 | Glaucous Gull | *Larus hyperboreus* | 4 |
| 247 | Great Black-backed Gull | *Larus marinus* | 3 |
| 248 | Sabine's Gull | *Xema sabini* | 2 |
| 249 | Black-legged Kittiwake | *Rissa tridactyla* | 8 |
| 250 | Red-legged Kittiwake | *Rissa brevirostris* | 2 |
| 251 | Ross’s Gull | *Rhodostethia rosea* | 1 |
| 252 | Ivory Gull | *Pagophila eburnea* | 1 |
| 253 | Gull-billed Tern | *Sterna nilotica* | 2 |
| 254 | Caspian Tern | *Sterna caspia* | 3 |
| 255 | Royal Tern | *Sterna maxima* | 4 |
| 256 | Elegant Tern | *Sterna elegans* | 5 |
| 257 | Sandwich Tern | *Sterna sandvicensis* | 8 |
| 258 | Common Tern | *Sterna hirundo* | 2 |
| 259 | Arctic Tern | *Sterna paradisaea* | 2 |
| 260 | Forster’s Tern | *Sterna forsteri* | 2 |
| 261 | Least Tern | *Sterna antillarum* | 2 |
| 262 | Aleutian Tern | *Sterna aleutica* | 5 |
| 263 | Bridled Tern | *Sterna anaethetus* | 2 |
| 264 | Sooty Tern | *Sterna fuscata* | 3 |
| 265 | Black Tern | *Chlidonias niger* | 2 |
| 266 | Brown Noddy | *Anous stolidus* | 1 |
| 267 | Black Skimmer | *Rynchops niger* | 2 |
| 268 | Dovekie | *Alle alle* | 4 |
| 269 | Common Murre | *Uria aalge* | 4 |
| 270 | Thick-billed Murre | *Uria lomvia* | 3 |
| 271 | Razorbill | *Alca torda* | 6 |
| 272 | Black Guillemot | *Cepphus grylle* | 4 |
| 273 | Pigeon Guillemot | *Cepphus columba* | 2 |
| 274 | Marbled Murrelet | *Brachyramphus marmoratus* | 3 |
| 275 | Kittlitz's Murrelet | *Brachyramphus brevirostris* | 7 |
| 276 | Xantus's Murrelet | *Synthliboramphus hypoleucus* | 1 |
| 277 | Ancient Murrelet | *Synthliboramphus antiquus* | 2 |
| 278 | Cassin’s Auklet | *Ptychoramphus aleuticus* | 1 |
| 279 | Parakeet Auklet | *Aethia psittacula* | 2 |
| 280 | Least Auklet | *Aethia pusilla* | 2 |
| 281 | Whiskered Auklet | *Aethia pygmaea* | 1 |
| 282 | Crested Auklet | *Aethia cristatella* | 1 |
| 283 | Rhinoceros Auklet | *Cerorhinca monocerata* | 4 |
| 284 | Atlantic Puffin | *Fratercula arctica* | 6 |
| 285 | Horned Puffin | *Fratercula corniculata* | 2 |
| 286 | Tufted Puffin | *Fratercula cirrhata* | 2 |
| 287 | Rock Pigeon | *Columba livia* | 3 |
| 288 | Red-billed Pigeon | *Patagioenas flavirostris* | 1 |
| 289 | Band-tailed Pigeon | *Patagioenas fasciata* | 2 |
| 290 | Eurasian Collared-Dove | *Streptopelia decaocto* | 2 |
| 291 | Spotted Dove | *Streptopelia chinensis* | 1 |
| 292 | White-winged Dove | *Zenaida asiatica* | 2 |
| 293 | Mourning Dove | *Zenaida macroura* | 7 |
| 294 | Inca Dove | *Columbina inca* | 6 |
| 295 | Common Ground-Dove | *Columbina passerina* | 8 |
| 296 | White-tipped Dove | *Leptotila verreauxi* | 3 |
| 297 | Budgerigar | *Melopsittacus undulatus* | 4 |
| 298 | Rose-ringed Parakeet | *Psittacula krameri* | 2 |
| 299 | Monk Parakeet | *Myiopsitta monachus* | 3 |
| 300 | Black-billed Cuckoo | *Coccyzus erythropthalmus* | 4 |
| 301 | Yellow-billed Cuckoo | *Coccyzus americanus* | 1 |
| 302 | Mangrove Cuckoo | *Coccyzus minor* | 2 |
| 303 | Greater Roadrunner | *Geococcyx californianus* | 1 |
| 304 | Smooth-billed Ani | *Crotophaga ani* | 4 |
| 305 | Groove-billed Ani | *Crotophaga sulcirostris* | 1 |
| 306 | Barn Owl | *Tyto alba* | 3 |
| 307 | Flammulated Owl | *Otus flammeolus* | 2 |
| 308 | Western Screech-Owl PS-1 | *Megascops kennicottii PS-1* | 3 |
| 309 | Western Screech-Owl PS-2 | *Megascops kennicottii PS-2* | 5 |
| 310 | Eastern Screech-Owl | *Megascops asio* | 9 |
| 311 | Great Horned Owl | *Bubo virginianus* | 6 |
| 312 | Snowy Owl | *Bubo scandiacus* | 4 |
| 313 | Northern Hawk Owl | *Surnia ulula* | 1 |
| 314 | Northern Pygmy-Owl | *Glaucidium gnoma* | 1 |
| 315 | Ferruginous Pygmy-Owl | *Glaucidium brasilianum* | 2 |
| 316 | Elf Owl | *Micrathene whitneyi* | 2 |
| 317 | Burrowing Owl | *Athene cunicularia* | 2 |
| 318 | Spotted Owl | *Strix occidentalis* | 7 |
| 319 | Barred Owl | *Strix varia* | 4 |
| 320 | Great Gray Owl | *Strix nebulosa* | 4 |
| 321 | Long-eared Owl | *Asio otus* | 6 |
| 322 | Short-eared Owl | *Asio flammeus* | 5 |
| 323 | Boreal Owl | *Aegolius funereus* | 3 |
| 324 | Northern Saw-whet Owl | *Aegolius acadicus* | 5 |
| 325 | Lesser Nighthawk | *Chordeiles acutipennis* | 2 |
| 326 | Common Nighthawk | *Chordeiles minor* | 3 |
| 327 | Common Pauraque | *Nyctidromus albicollis* | 10 |
| 328 | Common Poorwill | *Phalaenoptilus nuttallii* | 6 |
| 329 | Chuck-will’s-widow | *Caprimulgus carolinensis* | 2 |
| 330 | Whip-poor-will | *Caprimulgus vociferus* | 4 |
| 331 | Chimney Swift | *Chaetura pelagica* | 2 |
| 332 | Vaux’s Swift | *Chaetura vauxi* | 8 |
| 333 | White-throated Swift | *Aeronautes saxatalis* | 3 |
| 334 | Buff-bellied Hummingbird | *Amazilia yucatanensis* | 1 |
| 335 | Magnificent Hummingbird | *Eugenes fulgens* | 2 |
| 336 | Ruby-throated Hummingbird | *Archilochus colubris* | 5 |
| 337 | Black-chinned Hummingbird | *Archilochus alexandri* | 3 |
| 338 | Anna’s Hummingbird | *Calypte anna* | 4 |
| 339 | Costa’s Hummingbird | *Calypte costae* | 1 |
| 340 | Calliope Hummingbird | *Stellula calliope* | 4 |
| 341 | Broad-tailed Hummingbird | *Selasphorus platycercus* | 3 |
| 342 | Rufous Hummingbird | *Selasphorus rufus* | 4 |
| 343 | Allen’s Hummingbird | *Selasphorus sasin* | 2 |
| 344 | Ringed Kingfisher | *Ceryle torquata* | 3 |
| 345 | Belted Kingfisher | *Ceryle alcyon* | 4 |
| 346 | Green Kingfisher | *Chloroceryle americana* | 4 |
| 347 | Lewis’s Woodpecker | *Melanerpes lewis* | 5 |
| 348 | Red-headed Woodpecker | *Melanerpes erythrocephalus* | 2 |
| 349 | Acorn Woodpecker | *Melanerpes formicivorus* | 9 |
| 350 | Gila Woodpecker | *Melanerpes uropygialis* | 1 |
| 351 | Golden-fronted Woodpecker | *Melanerpes aurifrons* | 1 |
| 352 | Red-bellied Woodpecker | *Melanerpes carolinus* | 4 |
| 353 | Williamson's Sapsucker | *Sphyrapicus thyroideus* | 5 |
| 354 | Yellow-bellied Sapsucker | *Sphyrapicus varius* | 8 |
| 355 | Red-naped Sapsucker | *Sphyrapicus nuchalis* | 5 |
| 356 | Red-breasted Sapsucker | *Sphyrapicus ruber* | 6 |
| 357 | Ladder-backed Woodpecker | *Picoides scalaris* | 2 |
| 358 | Nuttall's Woodpecker | *Picoides nuttallii* | 4 |
| 359 | Downy Woodpecker | *Picoides pubescens* | 5 |
| 360 | Hairy Woodpecker | *Picoides villosus* | 9 |
| 361 | Arizona Woodpecker | *Picoides arizonae* | 1 |
| 362 | Red-cockaded Woodpecker | *Picoides borealis* | 2 |
| 363 | White-headed Woodpecker | *Picoides albolarvatus* | 7 |
| 364 | American Three-toed Woodpecker | *Picoides dorsalis* | 4 |
| 365 | Black-backed Woodpecker | *Picoides arcticus* | 3 |
| 366 | Northern Flicker | *Colaptes auratus* | 13 |
| 367 | Pileated Woodpecker | *Dryocopus pileatus* | 4 |
| 368 | Northern Beardless-Tyrannulet | *Camptostoma imberbe* | 2 |
| 369 | Olive-sided Flycatcher | *Contopus cooperi* | 2 |
| 370 | Western Wood-Pewee | *Contopus sordidulus* | 7 |
| 371 | Eastern Wood-Pewee | *Contopus virens* | 1 |
| 372 | Yellow-bellied Flycatcher | *Empidonax flaviventris* | 6 |
| 373 | Acadian Flycatcher | *Empidonax virescens* | 1 |
| 374 | Alder Flycatcher | *Empidonax alnorum* | 7 |
| 375 | Willow Flycatcher | *Empidonax traillii* | 4 |
| 376 | Least Flycatcher | *Empidonax minimus* | 4 |
| 377 | Hammond's Flycatcher | *Empidonax hammondii* | 5 |
| 378 | Gray Flycatcher | *Empidonax wrightii* | 2 |
| 379 | Dusky Flycatcher | *Empidonax oberholseri* | 1 |
| 380 | Pacific-slope Flycatcher | *Empidonax difficilis* | 5 |
| 381 | Cordilleran Flycatcher | *Empidonax occidentalis* | 2 |
| 382 | Buff-breasted Flycatcher | *Empidonax fulvifrons* | 2 |
| 383 | Black Phoebe | *Sayornis nigricans* | 3 |
| 384 | Eastern Phoebe | *Sayornis phoebe* | 1 |
| 385 | Say's Phoebe | *Sayornis saya* | 1 |
| 386 | Vermilion Flycatcher | *Pyrocephalus rubinus* | 5 |
| 387 | Dusky-capped Flycatcher | *Myiarchus tuberculifer* | 5 |
| 388 | Ash-throated Flycatcher | *Myiarchus cinerascens* | 5 |
| 389 | Great Crested Flycatcher | *Myiarchus crinitus* | 3 |
| 390 | Brown-crested Flycatcher | *Myiarchus tyrannulus* | 4 |
| 391 | Great Kiskadee | *Pitangus sulphuratus* | 4 |
| 392 | Sulphur-bellied Flycatcher | *Myiodynastes luteiventris* | 3 |
| 393 | Tropical Kingbird | *Tyrannus melancholicus* | 2 |
| 394 | Couch's Kingbird | *Tyrannus couchii* | 1 |
| 395 | Thick-billed Kingbird | *Tyrannus crassirostris* | 2 |
| 396 | Western Kingbird | *Tyrannus verticalis* | 1 |
| 397 | Gray Kingbird | *Tyrannus dominicensis* | 1 |
| 398 | Scissor-tailed Flycatcher | *Tyrannus forficatus* | 1 |
| 399 | Rose-throated Becard | *Pachyramphus aglaiae* | 2 |
| 400 | Loggerhead Shrike | *Lanius ludovicianus* | 6 |
| 401 | Northern Shrike | *Lanius excubitor* | 3 |
| 402 | White-eyed Vireo | *Vireo griseus* | 5 |
| 403 | Bell’s Vireo | *Vireo bellii* | 2 |
| 404 | Gray Vireo | *Vireo vicinior* | 1 |
| 405 | Cassin's Vireo | *Vireo cassinii* | 4 |
| 406 | Blue-headed Vireo | *Vireo solitarius* | 7 |
| 407 | Hutton’s Vireo | *Vireo huttoni* | 4 |
| 408 | Warbling Vireo PS-1 | *Vireo gilvus PS-1* | 3 |
| 409 | Warbling Vireo PS-2 | *Vireo gilvus PS-2* | 15 |
| 410 | Philadelphia Vireo | *Vireo philadelphicus* | 5 |
| 411 | Red-eyed Vireo | *Vireo olivaceus* | 9 |
| 412 | Yellow-green Vireo | *Vireo flavoviridis* | 10 |
| 413 | Black-whiskered Vireo | *Vireo altiloquus* | 2 |
| 414 | Gray Jay | *Perisoreus canadensis* | 2 |
| 415 | Steller’s Jay | *Cyanocitta stelleri* | 7 |
| 416 | Blue Jay | *Cyanocitta cristata* | 9 |
| 417 | Green Jay | *Cyanocorax yncas* | 2 |
| 418 | Florida Scrub-Jay | *Aphelocoma coerulescens* | 2 |
| 419 | Island Scrub-Jay | *Aphelocoma insularis* | 2 |
| 420 | Western Scrub-Jay PS-1 | *Aphelocoma californica PS-1* | 8 |
| 421 | Western Scrub-Jay PS-2 | *Aphelocoma californica PS-2* | 1 |
| 422 | Mexican Jay PS-1 | *Aphelocoma ultramarina PS-1* | 1 |
| 423 | Mexican Jay PS-2 | *Aphelocoma ultramarina PS-2* | 3 |
| 424 | Pinyon Jay | *Gymnorhinus cyanocephalus* | 6 |
| 425 | Clark’s Nutcracker | *Nucifraga columbiana* | 8 |
| 426 | Black-billed Magpie | *Pica hudsonia* | 3 |
| 427 | Yellow-billed Magpie | *Pica nuttalli* | 3 |
| 428 | American Crow | *Corvus brachyrhynchos* | 3 |
| 429 | Northwestern Crow | *Corvus caurinus* | 4 |
| 430 | Fish Crow | *Corvus ossifragus* | 2 |
| 431 | Chihuahuan Raven | *Corvus cryptoleucus* | 2 |
| 432 | Common Raven PS-1 | *Corvus corax PS-1* | 3 |
| 433 | Common Raven PS-2 | *Corvus corax PS-2* | 2 |
| 434 | Sky Lark | *Alauda arvensis* | 2 |
| 435 | Horned Lark | *Eremophila alpestris* | 3 |
| 436 | Purple Martin | *Progne subis* | 1 |
| 437 | Tree Swallow | *Tachycineta bicolor* | 8 |
| 438 | Violet-green Swallow | *Tachycineta thalassina* | 1 |
| 439 | Northern Rough-winged Swallow | *Stelgidopteryx serripennis* | 2 |
| 440 | Bank Swallow | *Riparia riparia* | 1 |
| 441 | Cliff Swallow | *Petrochelidon pyrrhonota* | 3 |
| 442 | Cave Swallow | *Petrochelidon fulva* | 2 |
| 443 | Barn Swallow | *Hirundo rustica* | 3 |
| 444 | Carolina Chickadee | *Poecile carolinensis* | 2 |
| 445 | Black-capped Chickadee | *Poecile atricapillus* | 5 |
| 446 | Mountain Chickadee PS-1 | *Poecile gambeli PS-1* | 3 |
| 447 | Mountain Chickadee PS-2 | *Poecile gambeli PS-2* | 4 |
| 448 | Mexican Chickadee | *Poecile sclateri* | 3 |
| 449 | Chestnut-backed Chickadee | *Poecile rufescens* | 7 |
| 450 | Boreal Chickadee | *Poecile hudsonicus* | 2 |
| 451 | Gray-headed Chickadee | *Poecile cincta* | 4 |
| 452 | Bridled Titmouse | *Baeolophus wollweberi* | 1 |
| 453 | Oak Titmouse | *Baeolophus inornatus* | 2 |
| 454 | Juniper Titmouse | *Baeolophus ridgwayi* | 2 |
| 455 | Tufted Titmouse | *Baeolophus bicolor* | 2 |
| 456 | Black-crested Titmouse | *Baeolophus atricristatus* | 2 |
| 457 | Verdin | *Auriparus flaviceps* | 3 |
| 458 | Bushtit PS-1 | *Psaltriparus minimus PS-1* | 2 |
| 459 | Bushtit PS-2 | *Psaltriparus minimus PS-2* | 2 |
| 460 | Red-breasted Nuthatch | *Sitta canadensis* | 7 |
| 461 | White-breasted Nuthatch | *Sitta carolinensis* | 5 |
| 462 | Pygmy Nuthatch | *Sitta pygmaea* | 5 |
| 463 | Brown-headed Nuthatch | *Sitta pusilla* | 2 |
| 464 | Brown Creeper | *Certhia americana* | 6 |
| 465 | Cactus Wren | *Campylorhynchus brunneicapillus* | 6 |
| 466 | Rock Wren | *Salpinctes obsoletus* | 3 |
| 467 | Canyon Wren | *Catherpes mexicanus* | 2 |
| 468 | Carolina Wren | *Thryothorus ludovicianus* | 5 |
| 469 | Bewick’s Wren PS-1 | *Thryomanes bewickii PS-1* | 4 |
| 470 | Bewick’s Wren PS-2 | *Thryomanes bewickii PS-2* | 2 |
| 471 | House Wren | *Troglodytes aedon* | 3 |
| 472 | Winter Wren PS-1 | *Troglodytes troglodytes PS-1* | 5 |
| 473 | Winter Wren PS-2 | *Troglodytes troglodytes PS-2* | 2 |
| 474 | Sedge Wren | *Cistothorus platensis* | 2 |
| 475 | Marsh Wren PS-1 | *Cistothorus palustris PS-1* | 5 |
| 476 | Marsh Wren PS-2 | *Cistothorus palustris PS-2* | 5 |
| 477 | American Dipper | *Cinclus mexicanus* | 9 |
| 478 | Golden-crowned Kinglet | *Regulus satrapa* | 7 |
| 479 | Ruby-crowned Kinglet | *Regulus calendula* | 6 |
| 480 | Arctic Warbler | *Phylloscopus borealis* | 2 |
| 481 | Blue-gray Gnatcatcher | *Polioptila caerulea* | 6 |
| 482 | California Gnatcatcher | *Polioptila californica* | 2 |
| 483 | Black-tailed Gnatchatcher | *Polioptila melanura* | 2 |
| 484 | Bluethroat | *Luscinia svecica* | 3 |
| 485 | Northern Wheatear | *Oenanthe oenanthe* | 2 |
| 486 | Eastern Bluebird | *Sialia sialis* | 6 |
| 487 | Western Bluebird | *Sialia mexicana* | 7 |
| 488 | Mountain Bluebird | *Sialia currucoides* | 5 |
| 489 | Townsend’s Solitaire | *Myadestes townsendi* | 7 |
| 490 | Veery | *Catharus fuscescens* | 5 |
| 491 | Gray-cheeked Thrush | *Catharus minimus* | 3 |
| 492 | Bicknell’s Thrush | *Catharus bicknelli* | 12 |
| 493 | Swainson’s Thrush | *Catharus ustulatus* | 8 |
| 494 | Hermit Thrush PS-1 | *Catharus guttatus PS-1* | 9 |
| 495 | Hermit Thrush PS-2 | *Catharus guttatus PS-2* | 7 |
| 496 | Wood Thrush | *Hylocichla mustelina* | 4 |
| 497 | American Robin | *Turdus migratorius* | 7 |
| 498 | Varied Thrush | *Ixoreus naevius* | 2 |
| 499 | Wrentit | *Chamaea fasciata* | 2 |
| 500 | Gray Catbird | *Dumetella carolinensis* | 7 |
| 501 | Northern Mockingbird | *Mimus polyglottos* | 2 |
| 502 | Sage Thrasher | *Oreoscoptes montanus* | 6 |
| 503 | Brown Thrasher | *Toxostoma rufum* | 6 |
| 504 | Curve-billed Thrasher PS-1 | *Toxostoma curvirostre PS-1* | 1 |
| 505 | Curve-billed Thrasher PS-2 | *Toxostoma curvirostre PS-2* | 2 |
| 506 | California Thrasher | *Toxostoma redivivum* | 1 |
| 507 | European Starling | *Sturnus vulgaris* | 9 |
| 508 | Common Myna | *Acridotheres tristis* | 2 |
| 509 | Eastern Yellow Wagtail | *Motacilla flava* | 3 |
| 510 | White Wagtail | *Motacilla alba* | 4 |
| 511 | American Pipit | *Anthus rubescens* | 2 |
| 512 | Sprague’s Pipit | *Anthus spragueii* | 1 |
| 513 | Bohemian Waxwing | *Bombycilla garrulus* | 2 |
| 514 | Cedar Waxwing | *Bombycilla cedrorum* | 5 |
| 515 | Phainopepla | *Phainopepla nitens* | 2 |
| 516 | Olive Warbler | *Peucedramus taeniatus* | 3 |
| 517 | Blue-winged Warbler | *Vermivora pinus* | 2 |
| 518 | Golden-winged Warbler | *Vermivora chrysoptera* | 2 |
| 519 | Tennessee Warbler | *Vermivora peregrina* | 3 |
| 520 | Orange-crowned Warbler | *Vermivora celata* | 4 |
| 521 | Nashville Warbler | *Vermivora ruficapilla* | 5 |
| 522 | Virginia’s Warbler | *Vermivora virginiae* | 3 |
| 523 | Colima Warbler | *Vermivora crissalis* | 1 |
| 524 | Lucy’s Warbler | *Vermivora luciae* | 2 |
| 525 | Northern Parula | *Parula americana* | 8 |
| 526 | Yellow Warbler | *Dendroica petechia* | 4 |
| 527 | Chestnut-sided Warbler | *Dendroica pensylvanica* | 4 |
| 528 | Magnolia Warbler | *Dendroica magnolia* | 3 |
| 529 | Cape May Warbler | *Dendroica tigrina* | 3 |
| 530 | Black-throated Blue Warbler | *Dendroica caerulescens* | 5 |
| 531 | Yellow-rumped Warbler | *Dendroica coronata* | 7 |
| 532 | Black-throated Gray Warbler | *Dendroica nigrescens* | 7 |
| 533 | Black-throated Green Warbler | *Dendroica virens* | 3 |
| 534 | Townsend’s Warbler | *Dendroica townsendi* | 6 |
| 535 | Hermit Warbler | *Dendroica occidentalis* | 5 |
| 536 | Blackburnian Warbler | *Dendroica fusca* | 3 |
| 537 | Yellow-throated Warbler | *Dendroica dominica* | 2 |
| 538 | Grace’s Warbler | *Dendroica graciae* | 3 |
| 539 | Pine Warbler | *Dendroica pinus* | 4 |
| 540 | Kirtland’s Warbler | *Dendroica kirtlandii* | 2 |
| 541 | Prairie Warbler | *Dendroica discolor* | 5 |
| 542 | Palm Warbler | *Dendroica palmarum* | 7 |
| 543 | Bay-breasted Warbler | *Dendroica castanea* | 4 |
| 544 | Blackpoll Warbler | *Dendroica striata* | 5 |
| 545 | Cerulean Warbler | *Dendroica cerulea* | 1 |
| 546 | Black-and-white Warbler | *Mniotilta varia* | 5 |
| 547 | American Redstart | *Setophaga ruticilla* | 5 |
| 548 | Prothonotary Warbler | *Protonotaria citrea* | 4 |
| 549 | Worm-eating Warbler | *Helmitheros vermivorus* | 2 |
| 550 | Swainson’s Warbler | *Limnothlypis swainsonii* | 1 |
| 551 | Ovenbird | *Seiurus aurocapillus* | 8 |
| 552 | Northern Waterthrush | *Seiurus noveboracensis* | 5 |
| 553 | Louisiana Waterthrush | *Seiurus motacilla* | 3 |
| 554 | Kentucky Warbler | *Oporornis formosus* | 1 |
| 555 | Connecticut Warbler | *Oporornis agilis* | 1 |
| 556 | Mourning Warbler | *Oporornis philadelphia* | 4 |
| 557 | MacGillivray’s Warbler | *Oporornis tolmiei* | 2 |
| 558 | Common Yellowthroat | *Geothlypis trichas* | 7 |
| 559 | Hooded Warbler | *Wilsonia citrina* | 2 |
| 560 | Wilson’s Warbler | *Wilsonia pusilla* | 6 |
| 561 | Canada Warbler | *Wilsonia canadensis* | 3 |
| 562 | Red-faced Warbler | *Cardellina rubrifrons* | 1 |
| 563 | Painted Redstart | *Myioborus pictus* | 1 |
| 564 | Yellow-breasted Chat | *Icteria virens* | 2 |
| 565 | Hepatic Tanager | *Piranga flava* | 1 |
| 566 | Summer Tanager | *Piranga rubra* | 3 |
| 567 | Scarlet Tanager | *Piranga olivacea* | 3 |
| 568 | Western Tanager | *Piranga ludoviciana* | 2 |
| 569 | White-collared Seedeater | *Sporophila torqueola* | 2 |
| 570 | Olive Sparrow | *Arremonops rufivirgatus* | 2 |
| 571 | Green-tailed Towhee | *Pipilo chlorurus* | 2 |
| 572 | Spotted Towhee | *Pipilo maculatus* | 7 |
| 573 | Eastern Towhee | *Pipilo erythrophthalmus* | 3 |
| 574 | Canyon Towhee | *Pipilo fuscus* | 2 |
| 575 | California Towhee | *Pipilo crissalis* | 2 |
| 576 | Abert’s Towhee | *Pipilo aberti* | 1 |
| 577 | Cassin’s Sparrow | *Aimophila cassinii* | 4 |
| 578 | Rufous-crowned Sparrow | *Aimophila ruficeps* | 2 |
| 579 | Tree Sparrow | *Spizella arborea* | 8 |
| 580 | Chipping Sparrow | *Spizella passerina* | 8 |
| 581 | Clay-colored Sparrow | *Spizella pallida* | 5 |
| 582 | Brewer’s Sparrow | *Spizella breweri* | 9 |
| 583 | Field Sparrow | *Spizella pusilla* | 4 |
| 584 | Black-chinned Sparrow | *Spizella atrogularis* | 1 |
| 585 | Vesper Sparrow | *Pooecetes gramineus* | 2 |
| 586 | Lark Sparrow | *Chondestes grammacus* | 2 |
| 587 | Black-throated Sparrow | *Amphispiza bilineata* | 6 |
| 588 | Sage Sparrow | *Amphispiza belli* | 5 |
| 589 | Lark Bunting | *Calamospiza melanocorys* | 5 |
| 590 | Savannah Sparrow | *Passerculus sandwichensis* | 9 |
| 591 | Grasshopper Sparrow | *Ammodramus savannarum* | 1 |
| 592 | Baird’s Sparrow | *Ammodramus bairdii* | 2 |
| 593 | Le Conte’s Sparrow | *Ammodramus leconteii* | 1 |
| 594 | Saltmarsh Sharp-tailed Sparrow | *Ammodramus caudacutus* | 3 |
| 595 | Seaside Sparrow | *Ammodramus maritimus* | 3 |
| 596 | Fox Sparrow | *Passerella iliaca* | 10 |
| 597 | Song Sparrow | *Melospiza melodia* | 5 |
| 598 | Lincoln’s Sparrow | *Melospiza lincolnii* | 7 |
| 599 | Swamp Sparrow | *Melospiza georgiana* | 6 |
| 600 | White-throated Sparrow | *Zonotrichia albicollis* | 7 |
| 601 | Harris’s Sparrow | *Zonotrichia querula* | 1 |
| 602 | White-crowned Sparrow | *Zonotrichia leucophrys* | 8 |
| 603 | Golden-crowned Sparrow | *Zonotrichia atricapilla* | 3 |
| 604 | Dark-eyed Junco | *Junco hyemalis* | 24 |
| 605 | Yellow-eyed Junco | *Junco phaeonotus* | 3 |
| 606 | McCown’s Longspur | *Calcarius mccownii* | 4 |
| 607 | Lapland Longspur | *Calcarius lapponicus* | 3 |
| 608 | Smith’s Longspur | *Calcarius pictus* | 2 |
| 609 | Chestnut-collared Longspur | *Calcarius ornatus* | 7 |
| 610 | Snow Bunting | *Plectrophenax nivalis* | 2 |
| 611 | McKay's Bunting | *Plectrophenax hyperboreus* | 1 |
| 612 | Northern Cardinal | *Cardinalis cardinalis* | 10 |
| 613 | Pyrrhuloxia | *Cardinalis sinuatus* | 1 |
| 614 | Rose-breasted Grosbeak | *Pheucticus ludovicianus* | 1 |
| 615 | Black-headed Grosbeak | *Pheucticus melanocephalus* | 7 |
| 616 | Blue Grosbeak | *Passerina caerulea* | 1 |
| 617 | Lazuli Bunting | *Passerina amoena* | 6 |
| 618 | Indigo Bunting | *Passerina cyanea* | 3 |
| 619 | Varied Bunting | *Passerina versicolor* | 4 |
| 620 | Painted Bunting | *Passerina ciris* | 3 |
| 621 | Dickcissel | *Spiza americana* | 2 |
| 622 | Bobolink | *Dolichonyx oryzivorus* | 6 |
| 623 | Red-winged Blackbird | *Agelaius phoeniceus* | 9 |
| 624 | Eastern Meadowlark PS-1 | *Sturnella magna PS-1* | 4 |
| 625 | Eastern Meadowlark PS-2 | *Sturnella magna PS-2* | 3 |
| 626 | Western Meadowlark | *Sturnella neglecta* | 4 |
| 627 | Yellow-headed Blackbird | *Xanthocephalus xanthocephalus* | 3 |
| 628 | Rusty Blackbird | *Euphagus carolinus* | 3 |
| 629 | Brewer’s Blackbird | *Euphagus cyanocephalus* | 6 |
| 630 | Common Grackle | *Quiscalus quiscula* | 4 |
| 631 | Boat-tailed Grackle | *Quiscalus major* | 6 |
| 632 | Great-tailed Grackle | *Quiscalus mexicanus* | 11 |
| 633 | Shiny Cowbird | *Molothrus bonariensis* | 2 |
| 634 | Bronzed Cowbird | *Molothrus aeneus* | 7 |
| 635 | Brown-headed Cowbird | *Molothrus ater* | 6 |
| 636 | Orchard Oriole | *Icterus spurius* | 2 |
| 637 | Hooded Oriole | *Icterus cucullatus* | 4 |
| 638 | Bullock's Oriole | *Icterus bullockii* | 5 |
| 639 | Spot-breasted Oriole | *Icterus pectoralis* | 1 |
| 640 | Altamira Oriole | *Icterus gularis* | 3 |
| 641 | Baltimore Oriole | *Icterus galbula* | 5 |
| 642 | Scott’s Oriole | *Icterus parisorum* | 1 |
| 643 | Brambling | *Fringilla montifringilla* | 2 |
| 644 | Gray-crowned Rosy-Finch | *Leucosticte tephrocotis* | 4 |
| 645 | Pine Grosbeak | *Pinicola enucleator* | 2 |
| 646 | Purple Finch | *Carpodacus purpureus* | 6 |
| 647 | Cassin’s Finch | *Carpodacus cassinii* | 5 |
| 648 | House Finch | *Carpodacus mexicanus* | 5 |
| 649 | Red Crossbill | *Loxia curvirostra* | 3 |
| 650 | White-winged Crossbill | *Loxia leucoptera* | 2 |
| 651 | Common Redpoll | *Carduelis flammea* | 2 |
| 652 | Hoary Redpoll | *Carduelis hornemanni* | 5 |
| 653 | Pine Siskin | *Carduelis pinus* | 6 |
| 654 | Lawrence’s Goldfinch | *Carduelis lawrencei* | 3 |
| 655 | American Goldfinch | *Carduelis tristis* | 8 |
| 656 | Evening Grosbeak | *Coccothraustes vespertinus* | 2 |
| 657 | House Sparrow | *Passer domesticus* | 4 |
| 658 | Eurasian Tree Sparrow | *Passer montanus* | 2 |

**Table S2.** COI barcode K2P distance between recently split species in the American Ornithologists’ Union Check List. Only splits within forms in U.S. and Canada are shown. (*) Barcode resolution not determined for splits due to lack of sequence data.

|  | Barcode  Resolution | Common Name | Species name | Interspecific  Distance |
| --- | --- | --- | --- | --- |
| 1 | distinct | Western Scrub-Jay  Florida Scrub-Jay | *Aphelocoma californica*  *Aphelocoma coerulescens* | 6.00 |
| 2 | distinct | Eastern Screech-Owl  Western Screech-Owl | *Megascops asio*  *Megascops kennicottii* | 5.92 |
| 3 | distinct | California Towhee  Canyon Towhee | *Pipilo crissalis*  *Pipilo fuscus* | 5.79 |
| 4 | distinct | American Golden Plover  Pacific Golden Plover | *Pluvialis dominica*  *Pluvialis fulva* | 4.82 |
| 5 | distinct | Baltimore Oriole  Bullock’s Oriole | *Icterus galbula*  *Icterus bullockii* | 4.33 |
| 6 | distinct | Oak Titmouse  Juniper Titmouse | *Baeolophus inornatus*  *Baeolophus ridgwayi* | 3.40 |
| 7 | distinct | Willow Flycatcher  Alder Flycatcher | *Empidonax traillii*  *Empidonax alnorum* | 3.22 |
| 8 | distinct | Yellow-bellied Sapsucker  Red-breasted Sapsucker | *Sphyrapicus varius*  *Sphyrapicus ruber* | 2.44 |
| 9 | distinct | Red-naped Sapsucker  Yellow-bellied Sapsucker | *Sphyrapicus nuchalis*  *Sphyrapicus varius* | 2.44 |
| 10 | distinct | Tropical Kingbird  Couch’s Kingbird | *Tyrannus melancholicus*  *Tyrannus couchii* | 2.19 |
| 11 | distinct | California Gnatcatcher  Black-Tailed Gnatcatcher | *Polioptila californica*  *Polioptila melanura* | 2.05 |
| 12 | distinct | Blue-Headed Vireo  Plumbeous Vireo | *Vireo solitarius*  *Vireo plumbeus* | 1.81 |
| 13 | distinct | Canada Goose  Cackling Goose | *Branta canadensis*  *Branta hutchinsii* | 1.62 |
| 14 | distinct | Dusky Grouse  Sooty Grouse | *Dendragapus obscurus*  *Dendragapus fuliginosus* | 1.25 |
| 15 | distinct | Western Scrub-Jay  Island Scrub-Jay | *Aphelocoma californica*  *Aphelocoma islandica* | 1.14 |
| 16 | distinct | Pacific Slope Flycatcher  Cordilleran Flycatcher | *Empidonax difficilis*  *Empidonax occidentalis* | 0.96 |
| 17 | distinct | Yellow-Green Vireo  Red-eyed Vireo | *Vireo flavoridis*  *Vireo olivaceus* | 0.91 |
| 18 | distinct | Gray-cheeked Thrush  Bicknell’s Thrush | *Catharus minimus*  *Catharus bicknelli* | 0.88 |
| 19 | distinct | Tufted Titmouse  Black-crested Titmouse | *Baelophus bicolor*  *Baelophus atricristatus* | 0.88 |
| 20 | distinct | Greater Sage Grouse  Gunnison’s Sage Grouse | *Centrocercus minimus*  *Centrocercus urophasianus* | 0.77 |
| 21 | distinct | Eastern Towhee  Spotted Towhee | *Pipilo erythrophthalmus*  *Pipilo maculatus* | 0.34 |
| 22 | overlap | Great-Tailed Grackle  Boat-tailed Grackle | *Quiscalis mexicanus*  *Quiscalis major* | 0 |
| 23 | overlap | Herring Gull  Thayer’s Gull | *Larus argentatus*  *Larus thayeri* | 0 |
| 24 | overlap | Western Grebe  Clark’s Grebe | *Aechmophorus occidentalis*  *Aechmophorus clarkii* | 0 |
| 25 | * | Western Gull  Yellow-footed Gull | *Larus occidentalis*  *Larus livens* | -- |
| 26 | * | Gray-crowned Rosy Finch  Black-crowned Rosy Finch  Brown-capped Rosy Finch | *Leucosticte tephrocotis*  *Leucosticte atrata*  *Leucosticte australis* | -- |
| 27 | * | Northern Flicker  Gilded Flicker | *Colaptes auratus*  *Colaptes chrysoides* | -- |
| 28 | * | Plumbeous Vireo  Cassin’s Vireo | *Vireo plumbeus*  *Vireo cassinii* | -- |
| 29 | * | Saltmarsh Sharp-tailed Sparrow  Nelson’s Sharp-tailed Sparrow | *Ammodramus caudacutus*  *Ammodramus nelsoni* | -- |
